# Chemical Inhibitors Targeting the Histone Lysine Demethylase Families with Potential for Drug Discovery

**DOI:** 10.3390/epigenomes7010007

**Published:** 2023-03-11

**Authors:** Nando Dulal Das, Hideaki Niwa, Takashi Umehara

**Affiliations:** Laboratory for Epigenetics Drug Discovery, RIKEN Center for Biosystems Dynamics Research, 1-7-22 Suehiro-cho, Tsurumi, Yokohama 230-0045, Japan

**Keywords:** chromatin, epigenetics, gene expression, post-translational modification, histone demethylation

## Abstract

The dynamic regulation of histone methylation and demethylation plays an important role in the regulation of gene expression. Aberrant expression of histone lysine demethylases has been implicated in various diseases including intractable cancers, and thus lysine demethylases serve as promising therapeutic targets. Recent studies in epigenomics and chemical biology have led to the development of a series of small-molecule demethylase inhibitors that are potent, specific, and have in vivo efficacy. In this review, we highlight emerging small-molecule inhibitors targeting the histone lysine demethylases and their progress toward drug discovery.

## 1. Introduction

Genes are expressed when genetic information from DNA is transcribed into RNA under the control of proteins that bind to specific DNA sequences. In both prokaryotes and eukaryotes, part of the mechanism that maintains the specificity of the gene expression is likely to be the positive feedback of the DNA-binding transcription factors [1,2], but in eukaryotes, there is an additional specific regulatory mechanism for gene expression; that is, the formation of chromatin structures with nucleosomes as the compaction unit [3,4]. The condensed state of chromatin is regulated by post-translational modifications (PTMs) of the core histone proteins, the major components of the nucleosome [5].

Core histones consist of four types, H2A, H2B, H3, and H4, and specific residues in the N-terminal tail of each are the major targets of PTMs including methylation, acetylation, phosphorylation, and ubiquitination [5,6,7,8,9]. These PTMs ultimately control the accessibility of the transcription machinery to DNA, thereby qualitatively and quantitatively regulating gene expression in the eukaryotic genome [5,10]. Of these modifications, the methylation of many lysine residues in the N-terminal tail of histone H3 (K4, K9, K27, K36) and H4K20 as well as some of the lysine residues in the core region such as H3K56 and H3K79, is a major regulator of gene expression [11]. The methylation of lysine residues has three different states: monomethyl (me1), dimethyl (me2), and trimethyl (me3), and these minute differences in methylation often have different regulatory significance. The methylation state of histones leads to chromatin opening or condensation and the subsequent activation or repression of transcription in various ways, depending on the residue that is methylated [12]. In addition, genome-wide studies suggest that the degree of methylation events and their position within the genome have important roles and could have specific consequences on chromatin states. For example, 11 different gene promoter states can be defined in human cells by different patterns of H3K4me1/H3K4me2/H3K4me3 or H3K79 methylation, and H4K20me1 or promoter acetylation [13,14].

Histone lysine methylation is reversibly modulated by histone lysine (K) methyltransferases (KMTs or HMTs) and lysine demethylases (KDMs). Lysine methylation is usually considered as a stable mark, and earlier, it was believed that histone lysine methylation is irreversible and can only be erased upon histone exchange or during DNA replication [11,15]. Following the discovery of lysine-specific demethylase 1 (LSD1/KDM1A), which catalyzes the demethylation of H3K4me1 and H3K4me2 [16], other lysine demethylases have been identified, and based on their catalytic functions, are classified into two major subgroups. The first family of lysine demethylases is composed of two members: KDM1A (also known as LSD1 [16]) and KDM1B (also known as LSD2 [17]), which can demethylate mono- and di-methyl-lysine residues. The second lysine demethylase family contains the Jumonji C (JmjC) domain, which can demethylate all three methyl-marks [18].

Aberrations in KMTs or KDMs are involved in the regulation of various diseases through the control of chromatin-related processes such as gene transcription and genome stability. Mutations or changes in gene expression involving the KMTs are often associated with diseases [19]. Dysregulation of several KDMs has also been implicated in diseases including cancer and inflammation [15,20]. The histone methylation states (e.g., Kme0 to Kme3) can be linked to unique biological activities [21] and the potential signaling mechanisms and biological pathways involved in diseases for each KMT family member have been summarized in recent reviews [20,22,23]. Therefore, this review focused on the lysine demethylase family.

Important feature of epigenomic drug discovery is that proteins acting on the epigenome often have either enzyme active centers that catalyze modification and de-modification or intramolecular concavities that selectively recognize specific modifications. Therefore, in many cases, their structure and function can be controlled by small molecular compounds, and chemical inhibitors of lysine demethylases have garnered interest as potential therapeutic agents against cancer and other diseases. In this review, we introduce the functions of each member of the lysine demethylase families and list the small molecular inhibitors of these families that have therapeutic potential in diseases.

## 2. Inhibitors of FAD–Containing Lysine Demethylases

**Figure 1:**
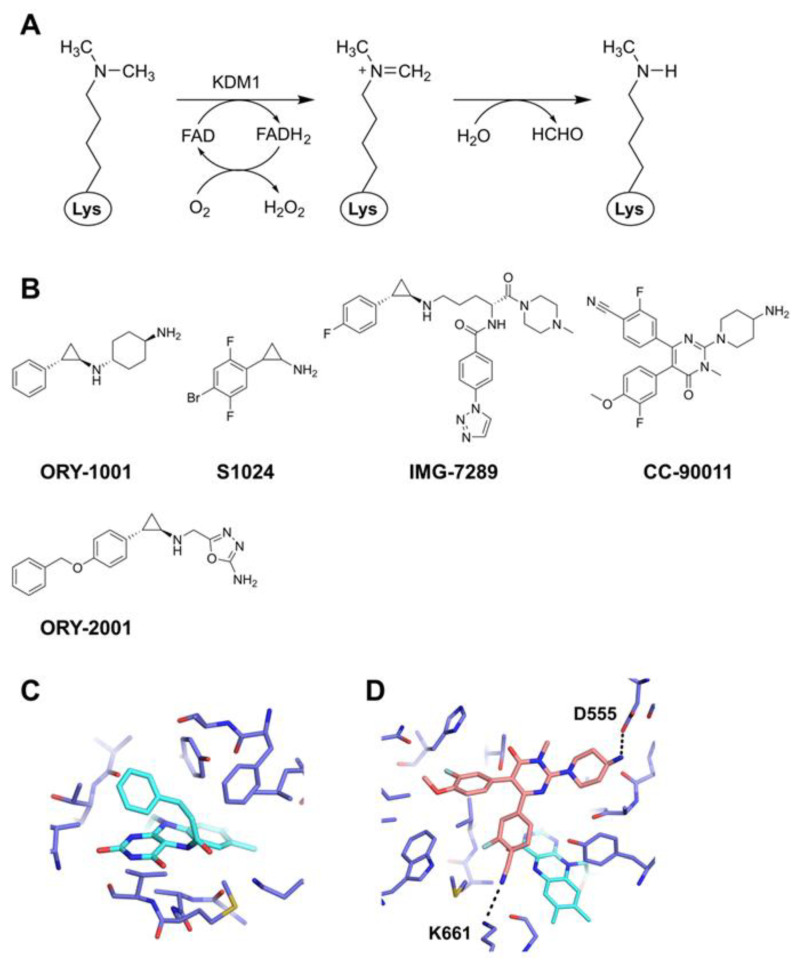
Inhibitors of the FAD–containing lysine demethylases. (**A**) Mechanism of the demethylation by KDM1A using FAD as a cofactor. (**B**) Representative inhibitors. (**C**) Structure of *trans*-(1R,2S)-2-phenylcyclopropylamine (PDB ID: 2XAJ) bound to KDM1A [37]. The adduct structure formed by this compound and FAD was assumed to be the same as the one formed by ORY-1001. The inhibitor–FAD adduct and KDM1A are colored in cyan and blue, respectively. The residues close to the adduct are drawn in sticks. (**D**) Structure of CC-90011 bound to KDM1A (PDB ID: 6W4K) [28]. The inhibitor and KDM1A are colored orange and blue, respectively, and FAD is colored cyan. The residues close to the adduct are drawn in sticks. Hydrogen bonds are shown by black dashed lines.

In humans, the flavin adenine dinucleotide (FAD)–containing lysine demethylases include KDM1A and KDM1B, which demethylate monomethyl and dimethyl methylated lysines (Kme1, Kme2) [24]. The KDM1 family enzymes are characterized by an amine oxidase-like domain that is responsible for the catalytic activity of demethylation [25]. Demethylation proceeds with the oxidation of the substrate methylated lysine by cofactor FAD, followed by an imine formation, and hydrolysis to demethylated lysine and formaldehyde (Figure 1A). Because a lone pair of electrons on the Nζ is necessary for the reaction, trimethylated lysine is not demethylated by this enzyme [26]. KDM1A demethylates monomethyl or dimethyl of histone H3K4 or H3K9 depending on the binding factor and isoform. Methylation of H3K4 and H3K9 generally acts to activate or repress gene transcription, respectively. Therefore, the regulatory mechanism of transcriptional activity by KDM1A is complex, and the genes subject to its regulation may vary from cell to cell.

Known KDM1A inhibitors include those that covalently inactivate FAD (e.g., ORY-1001 [27]) and those that non-covalently bind inside the enzyme active center (e.g., CC-90011 [28]) or peripheral to the binding pocket [24,29,30]. Clinical trials are underway for some of these inhibitors for the treatment of cancers including acute myeloid leukemia (AML) and small cell lung cancer (SCLC), solid tumors, myelofibrosis, non-Hodgkin’s lymphomas, and Alzheimer’s disease [31,32]. Chemical inhibition experiments have shown that KDM1A is not only involved in the control of cancer, but also in the modulation of metabolic properties by regulating the expression of genes involved in cellular energy expenditure and oxidative metabolism [33,34,35]. In an obese mouse model, systemic administration of a KDM1A inhibitor results in reduced food intake and body weight, and improves nonalcoholic fatty liver disease [36]. Representative KDM1A inhibitors include the examples described below and are shown in Figure 1B and Table 1.

ORY-1001 inhibits KDM1A enzymatic activities with an IC_50_ of 18 nM and induces AML differentiation [27]. Currently, it is in clinical trials for AML (NCT05546580) and SCLC (NCT05420636). ORY-1001 is a tranylcypromine-based inhibitor that inhibits KDM1A by forming a covalent bond with FAD, thereby arresting the demethylation reaction. The substituted amine group is dissociated during the reaction, as confirmed by mass spectrometry [27]. The structure of the resultant complex or adduct with FAD is expected to be the same as that of *trans*-(1R,2S)-2-phenylcyclopropylamine (PDB ID: 2XAJ; [37]; Figure 1C).

Recently, a dual KDM1 inhibitor S1024, which inhibits KDM1A and KDM1B at 0.094 and 8.4 μM, respectively, was developed [38] (Figure 1B). S1024 markedly increased the level of intracellular histone H3K4me2 more than the selective inhibitors against KDM1A, which should lead to further studies on the effects of a full blockade of FAD–containing KDMs on normal and diseased cells.

IMG-7289 is another irreversible tranylcypromine-based inhibitor for KDM1A (Figure 1B). In mouse models of myeloproliferative neoplasms, it normalizes blood cell counts, reduces spleen volumes, restores normal splenic architecture, and reduces bone marrow fibrosis. Importantly, it lowers the *Jak2^V617F^* mutant allele burden and improves survival [39]. Phase II clinical trials are underway for the treatment of myeloid-related diseases [40]. Additionally, a Phase IIb clinical trial is ongoing where IMG-7289 has shown promising results in reducing platelets and improving symptoms in essential thrombocythemia patients who are resistant or intolerant to at least one standard treatment (NCT04254978), leading to plans for a Phase III study [41].

ORY-2001 is a brain-penetrant inhibitor of KDM1A and monoamine oxidase B (MAO-B). It is a tranylcypromine-based covalent inhibitor, having IC_50_ values of 101 nM for KDM1A and 73 nM for MAO-B. It improves memory deficit and behavior alterations in the senescence accelerated mouse model, and social avoidance in the rat rearing isolation model [42]. It has been tested in a Phase IIa clinical trial for mild to moderate Alzheimer’s disease (NCT03867253) [31].

CC-90011 was developed as a non-covalent-type KDM1A inhibitor. CC-90011 inhibits KDM1A with an IC_50_ of 0.3 nM and induces cellular differentiation in the AML and SCLC cell lines. The crystal structure of the KDM1A/CC-90011 complex (PDB ID: 6W4K) shows that the molecule binds to the catalytic pocket with the aminopiperidine moiety interacting with D555 and the benzonitrile in a hydrophobic pocket, with the nitrile forming a hydrogen bond with K661, a key residue in the demethylation reaction. The 2-F-anisole ring is also in a hydrophobic pocket (Figure 1D) [28]. A Phase I clinical trial is underway for the treatment of patients with advanced or unresectable solid tumors including neuroendocrine neoplasms and relapsed/refractory non-Hodgkin lymphoma (NCT02875223) [43].

In addition to the demethylase activity, KDM1A interacts with transcription factors that contain an N-terminal Snail/growth factor independent 1 (GFI1) (SNAG) domain such as GFI1, GFI1B, or insulinoma-associated protein 1 (INSM1), which has a homologous amino acid sequence with that of histone H3 [44,45,46,47,48]. In AML, GFI1B interaction with KDM1A recruits KDM1A and corepressor complexes to their cognate genome-binding sites such as GFI1B target enhancers, thus repressing their activity. The drug-mediated disruption of a KDM1A–GFI1B complex induces the activation of GFI1B-target genes and is sufficient to block AML proliferation [48]. Similarly, in SCLC cells, KDM1A interacts with INSM1 and GFI1B to facilitate neuroendocrine-mediated transcription and cell proliferation [47]. Therefore, the critical role of the tranylcypromine-based KDM1A inhibitors in the treatment of AML [45,48] and SCLC [47] are presumably not only due to the inhibition of demethylation activity, but rather to the disruption of protein–protein interactions between KDM1A and interacted transcription factors.

Because tranylcypromine is an MAO inhibitor, tranylcypromine-based KDM1A inhibitors may react with FAD in MAO or other flavoenzymes. Therefore, the specificity of inhibition to KDM1A by the compound is important to avoid undesired off-target effects. In addition, disruption of the KDM1A–GFI1B complex by KDM1A inhibitors causes hematological toxicity such as thrombocytopenia. TAK-418, another tranylcypromine-based KDM1A inhibitor, avoids the disruption of KDM1A–GFI1B by forming a compact adduct with FAD through the degradation of an intermediate adduct form [49].

**Table 1:** Representative chemical inhibitors targeting the FAD–containing lysine demethylases.

Inhibitor	Target	Substrate	Potency	Application/Feature	Reference
ORY-1001	KDM1A	H3K4me2	0.0086 μM ^1,2^	Clinical trials for the treatment of AML (Phase Ib) and SCLC (Phase IIa)	[27]
S1024	KDM1A/1B	H3K4me2	0.094 μM ^1^	Chemical probe as a dual inhibitor of KDM1A and KDM1B for the study of H4K4me2 demethylation inhibition	[38]
IMG-7289	KDM1A	H3K4me2	0.25 μM ^1,2^	Clinical trials for the treatment of myeloid-related diseases (Phase II) and essential thrombocythemia (Phase IIb)	[39,40,41]
ORY-2001	KDM1A	H3K4me2	0.10 μM ^1^	Clinical trial (Phase IIa) for mild to moderate Alzheimer’s disease	[31,42]
CC-90011	KDM1A	H3K4me2	0.017 μM ^1,2^	Clinical trial (Phase I) for the treatment of neuroendocrine neoplasms and relapsed/refractory non-Hodgkin lymphoma	[28,43]

^1^ Half-maximal inhibition concentration (IC_50_) using horseradish peroxidase–coupled assay. ^2^ Assayed under the same experimental conditions [50].

## 3. Inhibitors of JmjC Domain–Containing Lysine Demethylases

**Figure 2:**
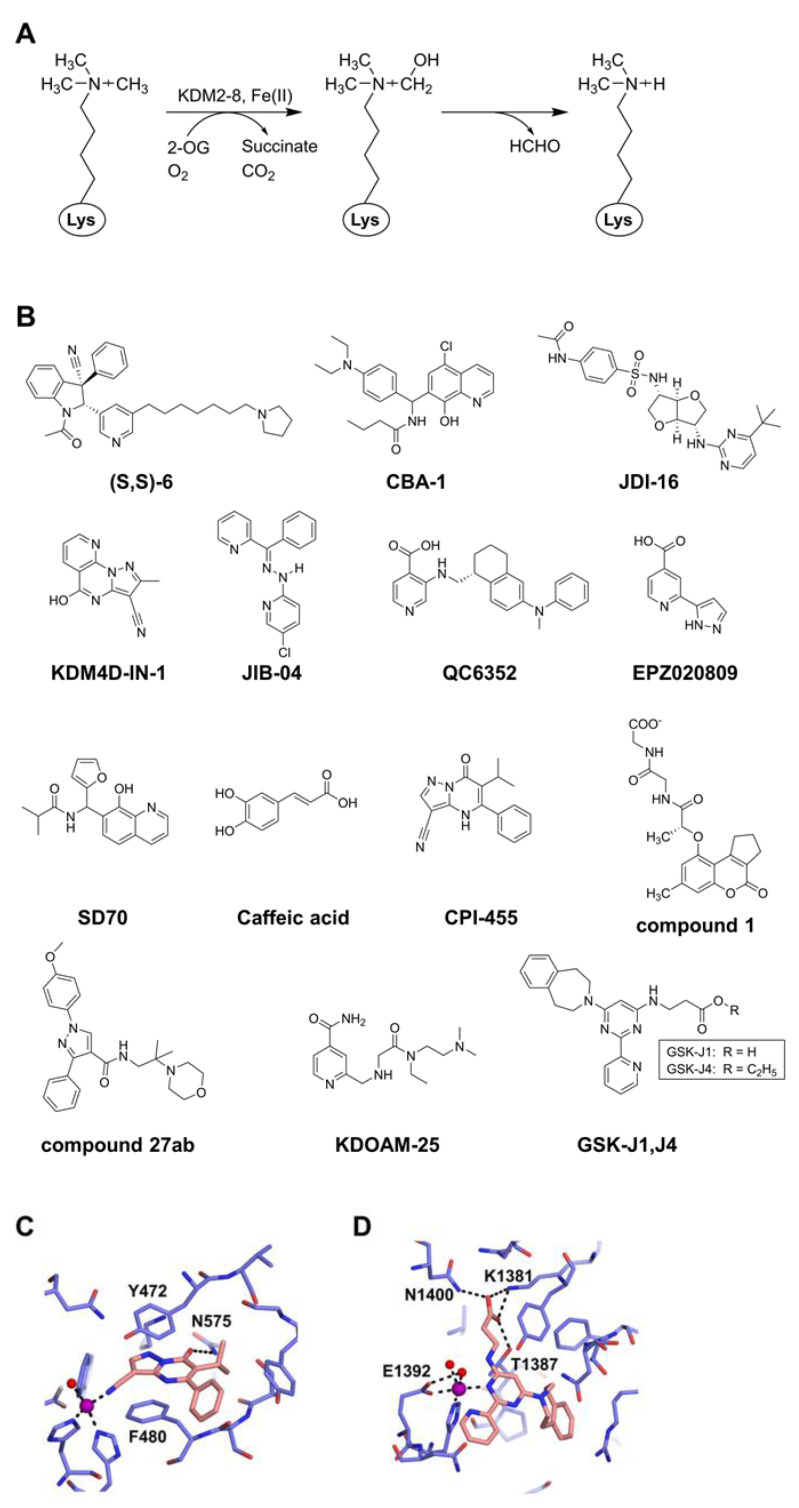
Inhibitors of the JmjC domain–containing lysine demethylases. (**A**) Mechanism of demethylation using 2-OG and Fe(II) as cofactors. (**B**) Representative inhibitors. (**C**) Structure of CPI-455 bound to KDM5A (PDB ID: 5CEH) [64]. The inhibitor and protein are colored orange and blue, respectively. The metal ion (Ni^2+^) and a coordinating water molecule are drawn in spheres, colored purple and red, respectively. Hydrogen bonds and metal coordination are shown by black dashed lines. (**D**) Structure of GSK-J1 bound to KDM6B (PDB ID: 4ASK) [65]. The inhibitor and protein are colored as in (**C**). The metal ion (Co^2+^) and coordinating water molecules are drawn in spheres, colored purple and red, respectively. Hydrogen bonds and metal coordination are shown as in (**C**).

A family of histone lysine demethylases distinct from the FAD–containing enzyme family is the enzyme family containing a catalytic JmjC domain, which demethylates monomethyl, dimethyl, and trimethyl methylated lysines (Kme1, Kme2, and Kme3) [51,52]. The enzymatic mechanism involves the oxidation of a methyl group using two co-factors, Fe(II) and 2-oxoglutarate (2-OG), which react with dioxygen to form a highly active oxoferryl (Fe(IV)=O) intermediate, and ultimately releases the methyl group from nitrogen in the form of formaldehyde [53] (Figure 2A). The JmjC domain–containing enzymes can be divided into seven subfamilies (i.e., KDM2 to KDM8). This family is known to include 33 proteins in humans, of which 18 are reported to function as histone demethylases [54,55]. Target residues for the demethylation of histone include H3K4 (KDM5 family), H3K9 (KDM3 family), H3K27 (KDM6 family), H3K36 (KDM2 and KDM4 families), and H4K20 (KDM7 family).

Due to the widespread upregulation across cancers and other diseases, the JmjC domain–containing family demethylases represent potentially good targets as epigenetic drugs for therapeutic purposes. Although many inhibitors of this family of demethylases are reported, the majority of them are 2-OG competitors that coordinate with Fe^2+^ ions in the catalytic center and sometimes compete with the histone substrate(s). However, most of these inhibitors are not specific only to the JmjC domain–containing demethylase, and the mechanisms of actions in cellular studies are poorly defined [56,57]. Moreover, the genome-wide effects of most of the inhibitors on methylated histones and in vivo specificities remain largely unclarified. In this section, we mainly focus on more potent inhibitors that have therapeutic potential (Figure 2B and Table 2).

### 3.1. Inhibitors of KDM2

KDM2 is the first JmjC domain–containing histone demethylase including two proteins (KDM2A and KDM2B), which demethylate H3K36me1/me2 [52]. Additionally, mammalian KDM2B can demethylate H3K4me3 [58]. In some cancers such as adenocarcinoma and lymphoma, KDM2B is highly expressed and acts as a putative proto-oncogene [59]. Many studies indicate that KDM2B has dual effects in cancer development. On the positive side, it stimulates the p15^Ink4b^ pathway [60], raises oxidative phosphorylation, and elevates KDM5A/MYC protein expression [61], resulting in increased cancer cell proliferation [62]. Alternatively, KDM2B hinders ribosomal RNA genes, MYC protein, and induces c-Fos ubiquitylation, leading to reduced cancer cell proliferation [62]. Due to the similarity in the catalytic domain of the KDM2 and KDM7 subfamilies, a potent and selective first-in-class inhibitor of KDM2A/7A, compound (S,S)-6, has been developed. Compound (S,S)-6 displays more than 75-fold selectivity toward KDM2A/7A versus other JmjC domain–containing lysine demethylases and has been demonstrated to reduce H3K36me2 demethylation within the cells [63].

### 3.2. Inhibitors of KDM3

This group of mammalian demethylases includes KDM3A (also known as JMJD1A) and two homologues of KDM3A: KDM3B (also known as JMJD1B) and KDM3C (also known JMJD1C). KDM3A is specific for the demethylation of H3K9me1/me2 [66] and has been shown to be important for spermatogenesis in mice [67,68]. It facilitates hypoxic gene expression, which enhances tumor growth in human renal and colon carcinoma cells [69] and multiple myeloma [70].

Two members of the KDM3 group, KDM3A and KDM3C, represent the most promising therapeutic targets for colorectal cancers (CRC) [71,72] and a subtype of acute leukemia, respectively [73]. KDM3A has been implicated in CRC progression via Wnt signaling where KDM3A coactivates downstream Wnt target genes including c-*Myc* and cyclin D1 [71,72]. A carboxamide-substituted benzhydryl amine, CBA-1, has been developed that acts as a KDM3A/3B inhibitor (mostly inhibiting KDM3A) and selectively induces elevated levels of H3K9me2, which in turn inhibits the Wnt targets (Auxin2, c-Myc, and Cyclin B1) and curtails in vitro CRC cell proliferation [72]. After the screening of thousands of compounds, the JmjC domain inhibitors JDI-4, JDI-12, and JDI-16, which share a common structural backbone, showed modest affinity with KDM3C and its family homologue KDM3B. In vivo demethylation assays indicated that compounds JDI-4 and JDI-12 could induce a global increase of H3K9 methylation. JDI-4 and JDI-12 can inhibit the growth of mixed lineage leukemia rearranged acute leukemia and other malignant hematopoietic cells, but not leukemia cells resistant to KDM3C depletion or cord blood cells. Importantly, compound JDI-16 exhibits a superior growth inhibition of malignant hematopoietic cells compared to JDI-4 or JDI-12 [73].

### 3.3. Inhibitors of KDM4

In mammals, there are four KDM4 demethylases that demethylate H3K9me2/me3 and H3K36me2/me3: KDM4A (also known as JMJD2A), KDM4B (also known as JMJD2B), KDM4C (also known as JMJD2C), and KDM4D (also known as JMJD2D, [51,74,75,76]. Several reports indicate that KDM4 family members are over-expressed in various cancers: KDM4A, KDM4B, and KDM4C are over-expressed in prostate cancer [74]; amplification of KDM4B is shown in medulloblastoma [77]; and KDM4C is required for the growth of breast carcinoma [78] and diffuse large B cell lymphoma [79]. These family members are involved in diverse biological pathways linked to cancer such as Akt-mTOR signaling for KDM4A, Wnt signaling for KDM4B, targeting pluripotency factors for KDM4C, and hypoxia-inducible factor 1 signaling for KDM4D [23]. Although the cellular functions of KDM4 demethylases are yet to be fully characterized, studies show that the demethylation of, for example, H3K9me3 at promoter regions, correlates with the activation of associated genes [80]. Given that these KDM4 demethylases, especially KDM4B and KDM4C, are involved in an array of cancers, targeting the catalytic activity of these demethylases could have therapeutic potential.

A recent study highlights the potential KDM4 inhibitors with their functions and therapeutic applications [81]. For instance, KDM4D-IN-1 is a specific inhibitor of KDM4D with an IC_50_ of 0.41 μM and shows anti-proliferative and anti-angiogenic effects on renal cell carcinoma cells both in vitro and in vivo [82]. IOX1 (also known as 5-c-8HQ, a derivative of 8-hydroxyquinoline) is another broad-spectrum inhibitor of the JmjC domain–containing demethylases (2-OG-dependent) including KDM3A, KDM4A, KDM4C, KDM4D, and KDM6B (also known as JMJD3) [83]. Subsequent in vitro and in vivo studies have shown that IOX1 acts as a potent inhibitor of KDM4D [84,85,86]. Another inhibitor, JIB-04, appears to chelate iron in the catalytic site and to disrupt histone substrate binding, thus inhibiting the demethylation activity of KDM4A, KDM4B, KDM4C, KDM4E, and KDM6B. JIB-04 shows anticancer activity and prolongs the survival of mice bearing orthotopic mammary tumors [87]. A later study indicated that along with chelating the metal center, JIB-04 disrupts the binding of O_2_ and histone substrates in the KDM4A active site by interacting with K241 and Y177 through hydrogen bonding [88]. By structure-based drug design, Celgene developed a novel KDM4 inhibitor, QC6352 (IC_50_ = 35−104 nM, for KDM4A–D), which potently suppresses the proliferation, sphere formation, and in vivo tumor growth of breast cancer and also reduces the tumor-initiating cell population in breast cancer [89]. A pan inhibitor of KDM4, TACH101, exhibits high inhibitory efficacy on all KDM4 isoforms (A-D) and demonstrates a potential therapy for gastrointestinal cancers. Currently, TACH101 is in a Phase I clinical trial for the treatment of gastrointestinal and high microsatellite instability metastatic colorectal cancers (NCT05076552) [90].

Another KDM4C-specific inhibitor, EPZ020809 (*K*_i_ = 31 nM), has been established that binds in a 2-OG-competitive fashion, where the nitrogen of the pyridine and a nitrogen of the pyrazole ring chelate the metal ion [91]. Regarding the specificity and potency of the in vivo antitumor activity, SD70, a derivative of 8-hydroxyquinoline, specifically inhibits KDM4C (IC_50_ = 30 μM) and reduces the tumor size in a mouse xenograft model of prostate cancer [92]. A clinical trial is ongoing to investigate the efficacy of caffeic acid (3,4-dihydroxycinnamic acid) for the treatment of esophageal cancer (NCT03070262) [93]. Caffeic acid was identified as an inhibitor of KDM4C and KDM6A (also known as ubiquitously transcribed X chromosome tetratricopeptide repeat protein, UTX) with IC_50_ values of 13.7 μM and 5.5 μM, respectively [94]. Specifically, KDM4C is upregulated in the tumor-initiating cells isolated from patient samples of esophageal squamous cell carcinoma, and caffeic acid treatment suppresses the demethylation activity of KDM4C [95]. Additionally, as a polyphenol, caffeic acid is found in coffee and it has been shown to be non-toxic, even at high doses of 0.5–1 g of daily consumption [96]. Furthermore, the inhibition of KDM4C with caffeic acid efficiently suppresses the human glioma xenograft tumors [97].

### 3.4. Inhibitors of KDM5

This demethylase family consists of KDM5A (also known as JARID1A), KDM5B (also known as JARID1B), KDM5C (also known as JARID1C), and KDM5D (also known as JARID1D), which catalyze the demethylation of H3K4me2/me3 [98,99,100]. Of the family members, KDM5B is overexpressed in an array of cancers including prostate [100], breast [101], and bladder carcinoma [102], and is also expressed in slow-growing cancer stem cells in melanoma [103]. KDM5C has been implicated in the repression of neuronal genes [104], and KDM5C knockdown in primary rat granule neurons hampers neuronal morphogenesis [99]. These findings suggest that KDM5 family members, especially KDM5B/5C, have therapeutic potential in cancers and neuronal disorders.

CPI-455 was reported as the first KDM5 inhibitor [64]. The crystal structure of the KDM5A/CPI-455 complex reveals that the inhibitor occupies the binding site of 2-OG, with the nitrile group interacting with the active site metal ion, the carbonyl oxygen forming a hydrogen bond with Nδ of N575, and the central aromatic core forming stacking with the side chains of Y472 and F480 [64] (Figure 2C). With the inhibition of KDM5B, CPI-455 reduces the stem-like properties of oral squamous cell carcinomas [105]. It also inhibits KDM5A. KDM5A is highly expressed in drug-resistant cells such as temozolomide (TMZ)-resistant glioblastoma cells, and CPI-455 is more effective in TMZ-resistant glioblastoma cells than in TMZ-native cells [106].

Compared with CPI-455, a derivative of cyclopenta[c]chromen named compound 1 exhibits higher potency against KDM5A (23.8 nM) and much higher selectivity for KDM5A over both KDM4A and other KDM5 family members (KDM5B and KDM5C) [107]. Compound 1 promotes the accumulation of p16 and p27 by inhibiting KDM5A-mediated H3K4me3 demethylation, leading to cell cycle arrest and the senescence of breast cancer cell lines [107]. Recently, one pyrazole derivative, compound 27 ab [1-(4-methoxyphenyl)-N-(2-methyl-2-morpholinopropyl)-3-phenyl-1H-pyrazole-4-carboxamide], has been discovered as a potent KDM5B inhibitor with an IC_50_ of 0.0244 μM [108]. A biological study revealed that compound 27 ab is a potent KDM5B inhibitor that accumulates H3K4me2/3 without affecting H3K4me1, H3K9me2/3, or H3K27me2 and can inhibit the proliferation and migration of a gastric cancer cell line [108]. In multiple myeloma, KDM5B acts as an oncogenic factor. Treatment with another KDM5 inhibitor, KDOAM-25M, in multiple myeloma cells inhibits cell proliferation and increases the global H3K4 methylation level at transcription sites [109]. However, to date, there are no KDM5-specific inhibitors in clinical trials.

### 3.5. Inhibitors of KDM6

In mammals, the KDM6 family consists of KDM6A, KDM6B, and UTY, which demethylate H3K27me2/me3 [110]. Mutated KDM6A has been implicated in multiple tumor types including multiple myeloma [111], renal cell carcinoma [111], and chronic myelomonocytic leukemia [112]. It is also overexpressed in breast cancer [113]. KDM6B is overexpressed in an array of cancers including lung, liver carcinoma, several hematological malignancies [114,115], and in primary Hodgkin’s lymphoma [116]. KDM6B is also involved in stress-induced gene transcription and is likely upregulated in activated macrophages [117].

One of the inhibitors developed with the most potential is GSK-J1, which is a specific inhibitor of KDM6B and KDM6A with an IC_50_ of 60 nM for KDM6B. GSK-J1 binds competitively to 2-OG, with its propanoic acid mimicking 2-OG binding and the pyridyl-pyrimidine biaryl chelating the active site metal [65] (Figure 2D). Such chelation is critical for the binding of the inhibitor very deep into the catalytic site of the substrate. Later, the potent cell-permeable analog GSK-J4 (the ethyl ester of GSK-J1) was developed. With the inhibition of KDM6B, GSK-J4 induces H3K27 methylation and shows potent antitumor efficacy in several cancers including glioma and leukemia [118,119], where GSK-J4 might be involved in the downregulation of cyclic-AMP response element–binding protein [120]. GSK-J4 suppresses the KDM6B-mediated proinflammatory response in macrophages [121], reduces tumor volume in mice xenografts of an ovarian cancer model [122], and reduces T-ALL xenograft growth in a mouse model [123]. These studies in mice xenograft models suggest that GSKJ1/4 could have therapeutic potential.

**Table 2:** Representative chemical inhibitors targeting the JmjC domain–containing lysine demethylases.

Inhibitor	Target	Substrate	Potency	Application	Reference
Compound (S,S)-6	KDM2A, KDM7A	H3K36me2	0.16 μM ^1^	Inhibits KDM2A-catalyzed demethylation in HeLa cells.	[63]
CBA-1	KDM3A/3B	H3K9me2	3.9 μM ^1^	Inhibits KDM3A overexpression in colon cancer cells and colon cancer organoids.	[72]
JDI-16	KDM3C	H3K9 methylation	0.82−6.12 μM ^1^	Represses multiple KDM3C-dependent leukemia cell lines and patient-derived primary leukemic cells; shows substantial growth inhibitory abilities against multiple hematopoietic malignant cells.	[73]
KDM4D-IN-1	KDM4D	H3K9 methylation	0.41 μM ^1^	Suppresses proliferation, induces apoptosis, and promotes angiogenesis of the renal cell carcinoma cells.	[82]
JIB-04	KDM4A/4B/4C/4E, KDM6B	H3K9me3	5.0 μM ^1^	Shows anti-cancer activity across several tumor types and in vivo mouse tumor xenografts; JIB-04 treatment induces cancer survival in an aggressive breast cancer model.	[87]
QC6352	KDM4A/4B/4C/4D	H3K9me3, H3K36me3	35−104 nM (KDM4A−4D) ^1^	Shows efficacy in patient-derived xenograft models of breast and colon cancers.	[89]
EPZ020809	KDM4C	H3K9 methylation	31 nM^2^	No information available.	
TACH101	A pan inhibitor of KDM4	No information available	0.004−0.072 µM (in gastric cancer cell lines) ^1^, 1–150 nM (in colorectal cancer cell lines) ^1^	A Phase I clinical trial is ongoing for the treatment of gastrointestinal and high microsatellite instability metastatic colorectal cancers.	[90]
SD70	KDM4C	H3K9me2	30 μM ^1^	Inhibits the proliferation of prostate cancer cells and shows inhibition of tumor growth in vivo.	[92]
Caffeic acid	KDM4C	H3K9me2/me3	13.7 μM ^1^	Effective against esophageal cancers; a Phase III clinical trial is ongoing for the treatment of esophageal squamous cell cancer; shows suppression of human glioma xenograft tumors.	[93,94,95,97]
CPI-455	KDM5A/5B	H3K4me3	10 nM ^1^	Attenuates the sphere formation of oral squamous cell carcinomas; effective against glioblastoma cells; effective against several KDM5-mediated drug-tolerant cancer cells such as HeLa, Colo829, and U2OS.	[64,105,106]
Cyclopenta[c] chromen derivative, compound 1	KDM5A	H3K4me3	23.8 nM ^1^	Shows efficacy against several KDM5A- overexpressing breast cancer cell lines such as MDA-MB-231, MCF-7, and MCF-10A.	[107]
Pyrazole derivative, compound 27 ab	KDM5B	H3K4me2/me3	0.0244 μM ^1^	Inhibits proliferation and migration abilities of MKN45, a gastric cancer cell line	[108]
KDOAM-25	KDM5A/5B/5C/5D	H3K4me3	71 nM (KDM5A), 19 nM (KDM5B), 69 nM (KDM5C and 5D) ^1^	Impairs proliferation of multiple myeloma cell.s	[109]
GSK-J1/J4	KDM6A/6B	H3K27me2/me3	60 nM^1^	Shows antitumor efficacy in several cancers, such as glioma and leukemia; effective to reduce tumor volume in mice xenograft models; suppresses KDM6B-mediated proinflammatory responses in macrophages.	[118,119,120,121,122,123]
Caffeic acid	KDM6A	Not studied	5.5 μM ^1^	No information available.	[94]

^1^ Half-maximal inhibition concentration (IC_50_). ^2^ Inhibition constant (*K*_i_).

### 3.6. Inhibitors of KDM7

The KDM7 family is also known as the PHF (plant homeodomain finger protein) family and consists of KDM7A (also known as JHDM1D), PHF2 (also known as JHDM1E), and PHF8 (also known as JHDM1F). KDM7A demethylates both H3K9me1/me2 and H3K27me1/me2, PHF2 demethylates H3K9me1, and PHF8 catalyzes the demethylation of H3K9me1/me2 [124,125,126]. Among the KDM7 demethylases, PHF8 has been involved in the regulation of X-linked mental retardation genes including KDM5C [127], and KDM7A has significant roles in the neuronal differentiation of mouse embryonic stem cells [128]. There is no potent and specific inhibitor reported for the KDM7 family of demethylases.

### 3.7. Inhibitors of KDM8

This family of lysine demethylases includes KDM8 (also known as JMJD5), JMJD6, NO66, and LOXL2. The substrate-specific catalytic activities of this family have not been confirmed in cellular studies; however, JMJD6 is reported to be a histone arginine demethylase (H4R3me1/me2 and H3R2me1/me2; [129]). Of the other members, NO66 catalyzes the demethylation of H3K4me2/me3 and H3K36me2/me3, which is overexpressed in non-small-cell lung cancer [130]. Although a couple of potent JMJD6 inhibitors such as SKLB325 [131] and 7p [132] have been discovered, an extended discussion on them was outside the scope of this review because they demethylate both lysine and arginine.

## 4. Conclusions

In this review, we introduced representative inhibitors of proteins belonging to the different categories of lysine demethylases in epigenetic drug discovery. The development of lysine demethylase inhibitors for therapy is challenging due to concerns about target selectivity, potential off-target effects, side effects, and toxicity. For instance, the JmjC domains in isoforms of the KDM4 family are structurally similar and shared molecular mechanisms make it difficult to design an isoform-specific inhibitor [133]. Moreover, the development of small-molecule inhibitors for the JmjC domain–containing lysine demethylases has been hindered by their polar 2-OG binding pocket and the lack of commercially available inhibitors [23]. Despite advances in understanding the catalytic domains of histone lysine demethylases, the role of non-catalytic domains is still limited and needs further study to fully understand the mechanisms controlling their demethylase activity. Because the lysine demethylase activity can affect transcriptional outputs in different ways depending on the cell types and target genes, the roles of lysine demethylases may vary among diseases [20]. The challenge in predicting the transcriptional and cellular outcomes of demethylase inhibition requires a fine balance to maximize the functional activities and to minimize the potential side effects. Epigenetic drug discovery often targets intractable diseases such as cancer, and to date, examples of practical applications of inhibitors of histone methyltransferases, histone deacetylases, and DNA methyltransferases are those for intractable cancer. Regarding lysine demethylases, inhibitors of KDM1A are in clinical trials for refractory cancers and Alzheimer’s disease, while inhibitors of KDM4 and KDM4C are only being studied for refractory cancers, and drug discovery in this field may be realized in the near future. Furthermore, an increasing number of clinical trials for epigenetic drug discovery have also been conducted for diseases other than cancer such as cardiovascular disease, diabetic kidney disease, and atherosclerosis [134,135,136]. Future epigenetic drug discovery is expected to develop therapeutics for an even wider range of diseases than is currently the case.

## Data Availability

Not applicable.
